# HVSMR-2.0: A 3D cardiovascular MR dataset for whole-heart segmentation in congenital heart disease

**DOI:** 10.1038/s41597-024-03469-9

**Published:** 2024-07-02

**Authors:** Danielle F. Pace, Hannah T. M. Contreras, Jennifer Romanowicz, Shruti Ghelani, Imon Rahaman, Yue Zhang, Patricia Gao, Mohammad Imrul Jubair, Tom Yeh, Polina Golland, Tal Geva, Sunil Ghelani, Andrew J. Powell, Mehdi Hedjazi Moghari

**Affiliations:** 1 A.A. Martinos Center for Biomedical Imaging, Massachusetts General Hospital, Boston, MA USA; 2 Computer Science and Artificial Intelligence Laboratory, Massachusetts Institute of Technology, Cambridge, MA USA; 3 Department of Electrical Engineering and Computer Science, Massachusetts Institute of Technology, Cambridge, MA USA; 4 Pediatric Surgical Research Laboratories, Massachusetts General Hospital, Harvard Medical School, Boston, MA USA; 5 Department of Pediatrics, Section of Cardiology, Children’s Hospital Colorado, Aurora, CO USA; 6 Department of Computer Science, University of Massachusetts Boston, Boston, MA USA; 7 Department of Biological Engineering, Massachusetts Institute of Technology, Cambridge, MA USA; 8 Feinberg School of Medicine, Northwestern University, Chicago, IL USA; 9 Department of Biochemistry and Molecular Genetics, Northwestern University, Chicago, IL USA; 10 School of Medicine, Indiana University, Indianapolis, IN USA; 11 Department of Computer Science, University of Colorado Boulder, Boulder, CO USA; 12 Department of Psychology, Ewha Womans University, Seoul, South Korea; 13 Department of Cardiology, Boston Children’s Hospital, Boston, MA USA; 14 Department of Pediatrics, Harvard Medical School, Boston, MA USA; 15 School of Medicine, The University of Colorado, Aurora, CO USA; 16 Department of Radiology, Children’s Hospital Colorado, Aurora, CO USA

**Keywords:** Three-dimensional imaging, Scientific data, Magnetic resonance imaging, Congenital heart defects

## Abstract

Patients with congenital heart disease often have cardiac anatomy that deviates significantly from normal, frequently requiring multiple heart surgeries. Image segmentation from a preoperative cardiovascular magnetic resonance (CMR) scan would enable creation of patient-specific 3D surface models of the heart, which have potential to improve surgical planning, enable surgical simulation, and allow automatic computation of quantitative metrics of heart function. However, there is no publicly available CMR dataset for whole-heart segmentation in patients with congenital heart disease. Here, we release the HVSMR-2.0 dataset, comprising 60 CMR scans alongside manual segmentation masks of the 4 cardiac chambers and 4 great vessels. The images showcase a wide range of heart defects and prior surgical interventions. The dataset also includes masks of required and optional extents of the great vessels, enabling fairer comparisons across algorithms. Detailed diagnoses for each subject are also provided. By releasing HVSMR-2.0, we aim to encourage development of robust segmentation algorithms and clinically relevant tools for congenital heart disease.

## Background & Summary

Cardiac malformations and changes in heart structure that are present at birth are collectively referred to as congenital heart disease (CHD)^1^. It is the leading cause of birth defect related deaths^2^. However, life expectancy is improving, leading to a growing population of adults with CHD who need ongoing care^3^. In CHD, vessels or chambers may be abnormally shaped (e.g., dilation), intracardiac connections may be atypical (e.g., double outlet right ventricle (DORV), in which the aorta connects to the right ventricle instead of the left ventricle), structure locations may be unexpected (e.g., the definitions of “left” and “right” ventricle are based on chamber characteristics and not location, so they may be swapped), and structures may be duplicated (e.g., two superior vena cavae) or missing (e.g., single ventricle and common atrium). Each CHD patient has a unique heart, with its own blend of original heart defects, prior surgical interventions, and transformations from long-term cardiac remodeling^4^.

Segmentation methods for CHD patients would be highly valuable^5^. Studies indicate that the greater appreciation of a patient’s unique anatomy provided by 3D patient-specific surface models may improve surgical planning and even prompt changes to surgical plans derived from imaging^6–10^. There are opportunities in surgical simulation, e.g., to simulate placement of valve implants, clips, baffles or Fontan conduits^11–14^. Moreover, automatic segmentation would facilitate quantitative metrics of cardiac function, such as chamber volumes, ejection fraction, and aortic dimensions^15,16^, which for such complex hearts are typically derived from hours of manual segmentation. However, this is a vulnerable, small, heterogeneous population unlikely to attract significant interest by industry^17^.

We describe the first public dataset for whole-heart segmentation from cardiovascular magnetic resonance (CMR) images from CHD patients. We use “whole-heart segmentation” to refer to the segmentation of 8 structures: the left ventricle (LV), right ventricle (RV), left atrium (LA), right atrium (RA), aorta (AO), pulmonary artery (PA), superior vena cava (SVC) and inferior vena cava (IVC). Some whole-heart segmentation datasets also include a label for the myocardium, which is not included here because it is much less important than the cardiac chambers and great vessels for the proposed clinical applications.

No currently available public dataset addresses all key factors. MM-WHS includes just 16 MR images from CHD patients, out of 60 CT images and 60 MR images^18^. The ImageCHD dataset contains CT images^19^. Babies and children with CHD undergo more surgery (and therefore imaging) to correct heart issues early in life. Avoiding ionizing radiation is particularly important for children, making CMR much more attractive. The 64 CMR scans released by Bidhendi *et al*.^20^ include LV/RV segmentations only. Finally, the first CHD MICCAI challenge, HVSMR, from our group contains 20 CMR images to be segmented into three classes: the global blood pool, myocardium/vessel walls, and background (http://segchd.csail.mit.edu)^21^. Relatively few methods had been developed for CHD before our challenge, which inspired many new works (e.g.^22–25^). However, a larger dataset of abnormal cases with a true whole-heart segmentation task is needed (see Arafati *et al*.^26^).

**Fig. 1:**
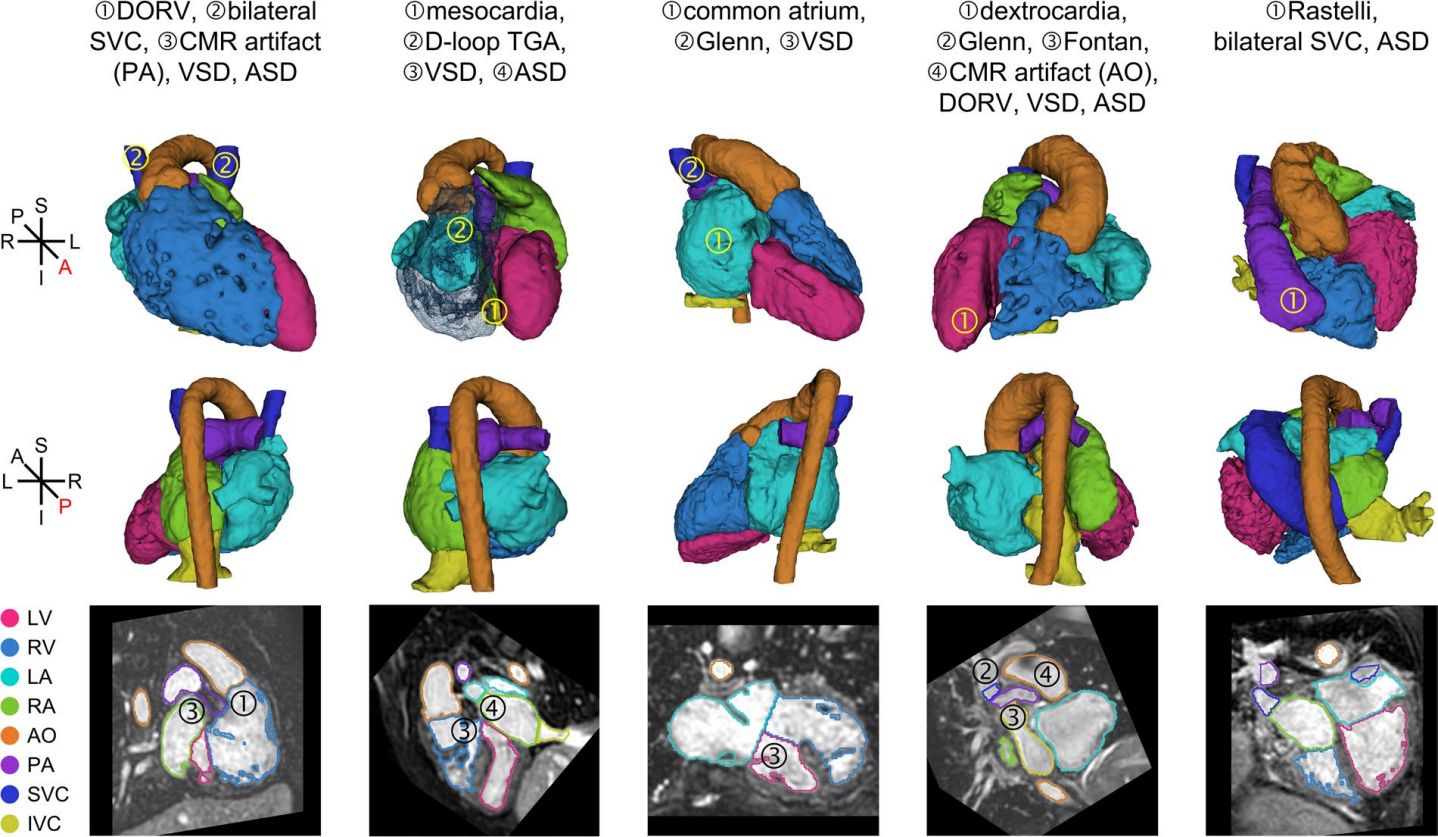
Example whole-heart segmentations in the HVSMR-2.0 dataset of cardiovascular magnetic resonance scans from patients with congenital heart disease (CHD). For definitions of each CHD subtype, see Table 1.

Some examples of our dataset are shown in Fig. 1. The HVSMR-2.0 dataset^27^ includes 60 CMR scans specifically chosen for their inclusion of diverse heart defects, including many not found in the datasets described above. Each image has a precise manual whole-heart segmentation. The main technical challenge is anatomical variability. CMR scans can also exhibit inhomogeneity and off-resonance artifacts, as well as large dark artifacts surrounding implanted stents. Furthermore, the valves and thin walls that separate neighboring structures are often beyond the imaging resolution.

We anticipate that HVSMR-2.0 will drive the development of innovative methodologies. These may also be useful to other applications with anatomical variability. Interactive segmentation may be particularly interesting, due to the challenges of this dataset, which may lead to new general-purpose algorithms to assist in dataset creation. It would also be beneficial to investigate the effectiveness of modern machine learning methods, such as diffusion and transformer-based models or topological priors, for CHD segmentation.

## Methods

### Ethical approval

The Institutional Review Board (IRB) at Boston Children’s Hospital approved this retrospective evaluation of imaging data and waived the requirement for written informed consent (IRB-P00011748). The IRB Chairs determined that the sharing of these 60 deidentified CMR scans and associated metadata via an open license is not inconsistent with the IRB’s approval of the original protocol or waiver of informed consent. The IRB has no objection to the sharing of these deidentified images and data.

### Subject selection

The dataset includes 60 images from patients with a wide variety of cardiac anatomies and defects, many of whom have undergone cardiac surgery for CHD. The distribution of diagnoses is given in Table 1. Subjects range in age from <1 to 52 years, with a mean age of 11.6 years and a median age of 10 years. There are 24 subjects aged 0–4 (neonates, babies, and toddlers), 25 subjects aged 5–18 (children) and 11 subjects aged 19 + (adults).

**Table 1:** Heart defects and surgeries.

	Description	Count	Percentage
**Mild**		**12**	**20%**
~Normal	Left: LA ↔ LV ↔ AO, Right: SVC/IVC ↔ RA ↔ RV ↔ PA	8	13%
Mild/Moderate Dilation	A chamber and/or vessel is mildly or moderately dilated	4	7%
**Moderate**		**11**	**18%**
VSD	Hole in the septal wall between the two ventricles	29	48%
ASD	Hole in the septal wall between the two atria	23	38%
DORV	AO ↔ RV and PA ↔ RV, always with VSD	22	37%
D-Loop TGA	AO ↔ RV and PA ↔ LV, with switched vessels	7	12%
S/P Arterial Switch for TGA	AO and PA are cut and reattached to the correct ventricle	1	2%
Bilateral SVC	Two SVCs, one on each side of the heart	9	15%
Severe Dilation	A chamber and/or vessel is severely dilated	4	7%
Tortuous Vessels	Vessels have abnormal twists and turns, due to arterial tortuousity syndrome or giant collaterals	2	3%
**Severe**		**37**	**62%**
Dextrocardia	Heart points to the right side	10	17%
Mesocardia	Heart points to the midline	4	7%
Inverted Ventricles	Labeled LV on right side of the body, labeled RV on left side	15	25%
Inverted Atria	Labeled LA on right side of the body, labeled RA on left side	7	12%
Left/Central IVC	IVC positioned on the left side or centrally	16	27%
Left/Central SVC	A single SVC positioned on the left side or centrally	6	10%
L-Loop TGA	AO ↔ RV and PA ↔ LV, with switched ventricles	3	5%
S/P Atrial Switch for TGA	Baffle connects SVC ↔ LV and IVC ↔ LV; return from pulmonary veins ↔ RV, AO ↔ RV and PA ↔ LV remain	1	2%
S/P Rastelli Procedure	Septal defect is patched; implanted baffle connects PA ↔ RV	2	3%
Single Ventricle	One ventricle, with any morphology, labeled as LV	6	10%
DILV	Large LV, small RV, AO ↔ LV, PA ↔ LV	4	7%
DI-DORV	Both atrioventricular valves ↔ RV, AO ↔ RV, PA ↔ RV	1	2%
Common Atrium	One atrium, labeled as LA	10	17%
S/P Glenn Procedure	SVC ↔ PA	24	40%
S/P Fontan Procedure	IVC ↔ PA, always S/P Glenn procedure	9	15%
**Coincident Variants**			
Heterotaxy	Abnormal left/right arrangement of the heart or other organs	13	22%
Superoinferior Ventricles	Ventricles sit one above the other	3	5%
PA Atresia or MPA Stump	Pulmonary valve is poorly developed or surgically closed	9	15%
S/P PA Banding	Implanted band to narrow the main PA trunk	7	12%
AO-PA Anastomosis	Damus-Kaye-Stansel anastomosis (proximal PA sewn to aorta)	4	7%
Marfan Syndrome	Connective tissue disorder, can cause abnormal chest anatomy	3	5%
CMR Artifact (AO)	Dark artifact in the AO	9	15%
CMR Artifact (PA)	Dark artifact in the PA	13	22%

Subjects can have multiple diagnoses, and are categorized as “mild”, “moderate” or “severe” according to the most serious defect in the case. Coincident variants are not used to categorize subjects. Prior conditions that have been previously corrected such that they are no longer visible in the scan are not included. However, hearts with multiple defects may deviate in anatomy from the descriptions below (e.g., patients with DORV who are S/P Glenn procedure have SVC ↔ PA and not PA ↔ RV). A dilated chamber or vessel is only noted if it is the sole diagnosis. The ↔ symbol indicates that two structures are connected, with blood flow between them. VSD = ventricular septal defect, ASD = atrial septal defect, DORV = double outlet right ventricle, TGA = transposition of the great arteries, DILV = double inlet left ventricle, DI-DORV = double inlet-double outlet right ventricle, S/P = status post (previous surgery).

The first 20 images come from the original HVSMR dataset, and we subsequently chose an additional 40 cases that encapsulate a wider range of CHD subtypes. We aimed to create a balanced dataset that samples the different heart defects and their combinations as uniformly as possible, so that any models trained using this dataset will be applicable to a wide range of defects. However, some imbalance is inevitable, since some defects are much more common than others (e.g., ventricular and atrial septal defects co-occur with many other heart defects).

The 20 original HVSMR images were chosen on a rolling basis, as acquired under standard clinical practice at Boston Children’s Hospital. The inclusion criteria for this study encompassed four key parameters. First, a prerequisite was high image quality, necessitating minimal bulk motion of patients; images displaying substantial patient motion were excluded. Second, signal homogeneity in both blood and myocardium was crucial, leading to the exclusion of images featuring significant signal inhomogeneity where the border between myocardium and blood was not visually identifiable. Third, the strength of off-resonance artifacts causing significant signal void in blood or myocardium was considered. Finally, the fourth criterion involved ensuring an adequate field of view, specifically encompassing the entire LV, RV, and great vasculature within the scan.

To choose the remaining 40 images, 3606 potential cases were identified by searching the written radiology reports at Boston Children’s Hospital for keywords indicating that a 3D CMR scan was acquired. Each radiology report includes standardized “cardiology codes”, which enumerate hundreds of different diagnoses, abnormalities in cardiac anatomy or function, and prior interventions. We identified codes pertaining to important heart defects and corrective surgeries of interest under the advice of a pediatric cardiologist (Su.G.), and manually selected 40 CMR studies with both (1) the codes required to create an overall dataset that was as balanced as possible, and (2) high image quality, as described above.

### Subject categorization

Each case was classified as having mild, moderate or severe anatomical malformations, under the advice of a pediatric cardiologist (Su.G.). Definitions of CHD subtypes and surgeries are provided in Table 1. *Mild:* roughly normal anatomy, prior CHD surgery with restoration of normal anatomy, and/or a mildly or moderately dilated chamber or vessel. *Moderate:* abnormal connectivity, septal defect, bilateral SVC, a severely dilated chamber or vessel, and/or congenital connective tissue disorder causing tortuous vessels. *Severe:* Heart malposition or situs inversus, L-loop TGA, single ventricle, common atrium, and/or major prior reconstructive surgery resulting in highly abnormal anatomy. There are 12 “mild” subjects, 11 “moderate” subjects, and 37 “severe” subjects. Note that these categories represent deviations from normal anatomy, and not the patient’s prognosis, and that “moderate” cases still have significant heart defects (e.g., DORV). Most subjects have a unique combination of heart defects and prior surgeries (ignoring coincident variants): 64% of moderate subjects and 95% of severe subjects.

### Image acquisition

All images capture a snapshot of the heart at high resolution, i.e., they depict a static heart and not a beating heart. 3D CMR images were acquired in an axial view on a clinical 1.5 T scanner (Philips Achieva) during clinical routine at Boston Children’s Hospital. Most images were acquired using a respiratory navigator technique during a free-breathing acquisition using a steady-state free precession (SSFP) pulse sequence, with prospective ECG gating to freeze cardiac motion and generate a static image of the heart. Intravenous gadolinium-based contrast agent (Ablavar (gadofoveset) or Gadovist) was used in many patients. Whether or not to use contrast was decided by the clinician. Since some institutions use contrast and some do not, it is helpful clinically that our dataset includes examples of both.

All images were manually cropped at the level of the chin to ensure that no facial features are present. As the images were originally acquired in a clinical environment, each image has a different size (481 × 410 × 171 on average, ranging from [256–720] × [120–607] × [90–528]). Each image has near-isotropic resolution (0.73 × 0.73 × 0.81 mm on average, ranging from [0.52–1.15] × [0.52–1.15] × [0.38–1.60] mm). The intensity range within the entire dataset is ~[0, 7500]. Imaging parameters ranged between: echo time 1.5–2.4 ms, repetition time 3.1–4.9 ms, flip angle 55–110°, bandwidth 540–1575 Hz.

### Image preprocessing

The images were manually cropped to a tight region around the heart. The acquired field of view is different for babies, children and adults, so this step ensures that the size of each anatomical structure within the cropped images is roughly the same for all ages, greatly simplifying model training. The cropped image sizes vary (150 × 193 × 154 on average, ranging from [83–273] × [95–322] × [77–220]). Each cropped image has the same resolution as the original.

All cropped images were normalized using a customized scheme. For each image, two mean intensities were estimated: one for the cardiac blood pool and one for the lungs. The estimated blood pool intensity was mapped to 0.8, the estimated lung intensity was mapped to 0.07, and a linear transfer function was used to rescale each image, yielding an intensity range within the entire dataset of ~[−0.1, 3.3]. Each cropped image was transformed into an approximate short-axis orientation, and we estimated the blood pool intensity by automatically extracting a slab of the cropped images that typically contains the ventricles, and using the Mean Shift Algorithm^28^ to find the peak of the intensity histogram that corresponds to the blood pool. Similarly, we estimated the typical lung intensity by extracting a slab in the upper portion of each transformed cropped image that typically contains the lungs only, and using the mode of the resulting intensity histogram.

### Ground truth segmentation

A detailed description of the protocol for segmenting each structure is provided in Table 2, which was created in close consultation with pediatric cardiologists at Boston Children’s Hospital and Children’s Hospital Colorado (T.G., Su.G., A.J.P, and J.R.). Note that some structures have required and/or optional areas. Many interfaces between structures have no clear intensity boundary. Valves are often too thin and fast-moving to be imaged, and some boundaries at septal defects or other junctions (e.g., SVC/RA or IVC/RA) are determined by the gross anatomy and not by any image gradients. To standardize the segmentation process, manual segmentations were performed so that interfaces separating different structures were approximately planar, unless a curved interface was clearly present. All segmentations were performed based on the 3D CMR image for each patient, without access to additional scans such as angiography, motion or flow information.

**Table 2:** Ground truth definitions of each cardiac structure and their optional zones.

**LV/RV**	The LV and RV are differentiated by considering chamber shape, surface smoothness, placement of the atrioventricular valves, and the radiology report. Note that it is possible for the anatomic LV to be on the right side of the body and the anatomic RV on the left side of the body. There are several single ventricle subtypes. In Double Inlet Left Ventricle (DILV) cases, there is a large LV, and the small outflow chamber is labeled as RV. In Double Inlet/Double Outlet Right Single Ventricle cases, the RV is large and there is a very small labeled LV. In Holmes cases, there is a single LV and the RV label is empty. For all other single ventricle hearts, the single ventricle is labeled as LV and the RV label is empty (we desired a consistent definition to increase the feasibility of automated segmentation via machine learning, as in our experience, trained networks often output a mixture of LV and RV labels within single ventricles).
**LV**	Typically bordered by the mitral valve, aortic valve, and/or VSD (if present). Includes the outflow portion. As is standard clinical practice at Boston Children’s Hospital and as advised by cardiologists, the papillary muscles are not included in order to create realistic 3D heart models for surgical planning.
**RV**	Typically bordered by the tricuspid valve, pulmonary valve, and/or VSD (if present). Includes the outflow portion. As is standard clinical practice at Boston Children’s Hospital and as advised by cardiologists, the trabeculations are not included in order to create realistic 3D heart models for surgical planning. For patients who have undergone a Glenn or Fontan procedure, the residual PA stump is labeled as RV. In the setting of significant RV hypertrophy, insufficient image resolution and noise may cause manual segmentations to be undersegmented.
**LA/RA**	We choose to label the chamber with attached pulmonary veins (PVs) as the LA, and the other chamber (if present) as the RA. If there is a common atrium, it is labeled as LA (RA is empty). However, like the LV and RV, the atria are clinically defined by anatomy, and not by pulmonary veins coming in. In particular, heterotaxy patients do not have a LA or a RA, but rather a “left-sided atrium” and a “right-sided atrium” based purely on their position in the thorax. However, we desired a more consistent definition that would be the same for both heterotaxy and non-heterotaxy patients, again based on our experience that trained networks can assign a mixture of LA and RA labels to a common atrium. In clinical practice, the assignment of left vs. right for heterotaxy patients may need to be manually edited following inference.
**LA**	Typically bordered by the mitral valve and/or ASD (if present). The ground truth includes the PVs attached to the LA until they branch. Any confluence between the PVs and the LA is also segmented as LA. The PVs can be optionally shorter. This was defined by manually cutting each PV to require its stump only.
**RA**	Typically bordered by the tricuspid valve, SVC insertion, IVC insertion, and/or ASD (if present).
**AO**	From the aortic valve through the ascending and descending aorta, until the most inferior level of the LV/RV/LA/RA/PA. In AO-PA anastomosis cases, the AO label includes the segment of the attached original PA. Includes the aortic ductus diverticulum if present. Can optionally continue to the bottom of the image.
**PA**	Typically includes the main PA trunk, from the pulmonary valve, and the left and right PA branches. The ground truth segmentations of the left and right branches have equal length, which is defined by the distance from the bifurcation point to behind the right upper lobe segmental branch. For Glenn/Fontan patients, the PA often consists of only the two branches. Effort was made to track through CMR inhomogeneity artifacts; if this was impossible then any disconnected segments were labeled as optional. The distal ~25% of each ground truth branch segmentation is optional. The left and right branches can optionally continue beyond the ground truth stopping point, until the point at which they split into their lower lobe segmental branches.
**SVC**	From the most superior axial slice at the level of its bifurcation into the brachiocephalic veins, down to its insertion into the attached atrium (angled according to atrium curvature) or the PA branches (Glenn/Fontan patients). A second SVC may be present (bilateral SVC). The superior ~25% in the ground truth is optional. Each SVC can optionally continue higher, through the brachiocephalic vein and internal jugular veins.
**IVC**	From its insertion into the attached atrium (angled according to atrium curvature) or the PA branches (Fontan patients: baffle included), down through the hepatic segment and subsequent branches of the hepatic veins. Rarely, has two connected components, depending on the insertion of the hepatics. The ground truth length was defined by identifying the level of the first bifurcation, counting down by 5% of the image height, and then dilating the pre-bifurcation segment to this level (so that branches are cut at an angle). The required segment was defined by repeating this using the lowest axial slice in which the IVC appeared round (i.e., above any branching) and counting by 2/3·5% of the image height. The IVC can optionally continue branching to the bottom of the image.

All of the tools we used to create the ground truth whole-heart segmentations are open source. All manual segmentations were performed using 3D Slicer (http://www.slicer.org)^29^, which includes helpful modules for manual painting (with or without an editable intensity range), island processing, logical operators, 3D surface model editing (e.g., using scissors) and smoothing.

#### Segmenting the 20 HVSMR images

The 20 HVSMR images already had ground truth manual segmentations of the blood pool and myocardium, which were created using manual painting in an approximate short-axis view. The main tool was 3D Slicer’s paint option with an editable intensity range, in which intensity thresholding is applied in the region under the paintbrush only, providing a more objective and precise way of determining the boundary between the blood pool and the neighboring myocardium or vessel/chamber wall. These segmentations had already been reviewed by an associate professor in pediatric radiology and cardiology (M.H.M.) and a pediatric cardiologist (A.J.P).

For our purposes, the blood pool label had to be further split into 8 compartments (LV, RV, LA, RA, AO, PA, SVC, IVC). Trained raters (graduate and undergraduate students in medical image analysis, computer science and the life sciences) manually divided each blood pool into its constituent parts by creating a 3D blood pool surface model from the existing HVSMR segmentation, dropping fiducial landmarks onto the surface at the interfaces between structures, and fitting local separating planes. Each rater was trained in a specific subtask (e.g., mitral valve annotation) to avoid inter-observer variability. The annotations from the different raters were combined to create a single whole-heart segmentation.

#### Segmenting the 40 new images

**Fig. 2:**
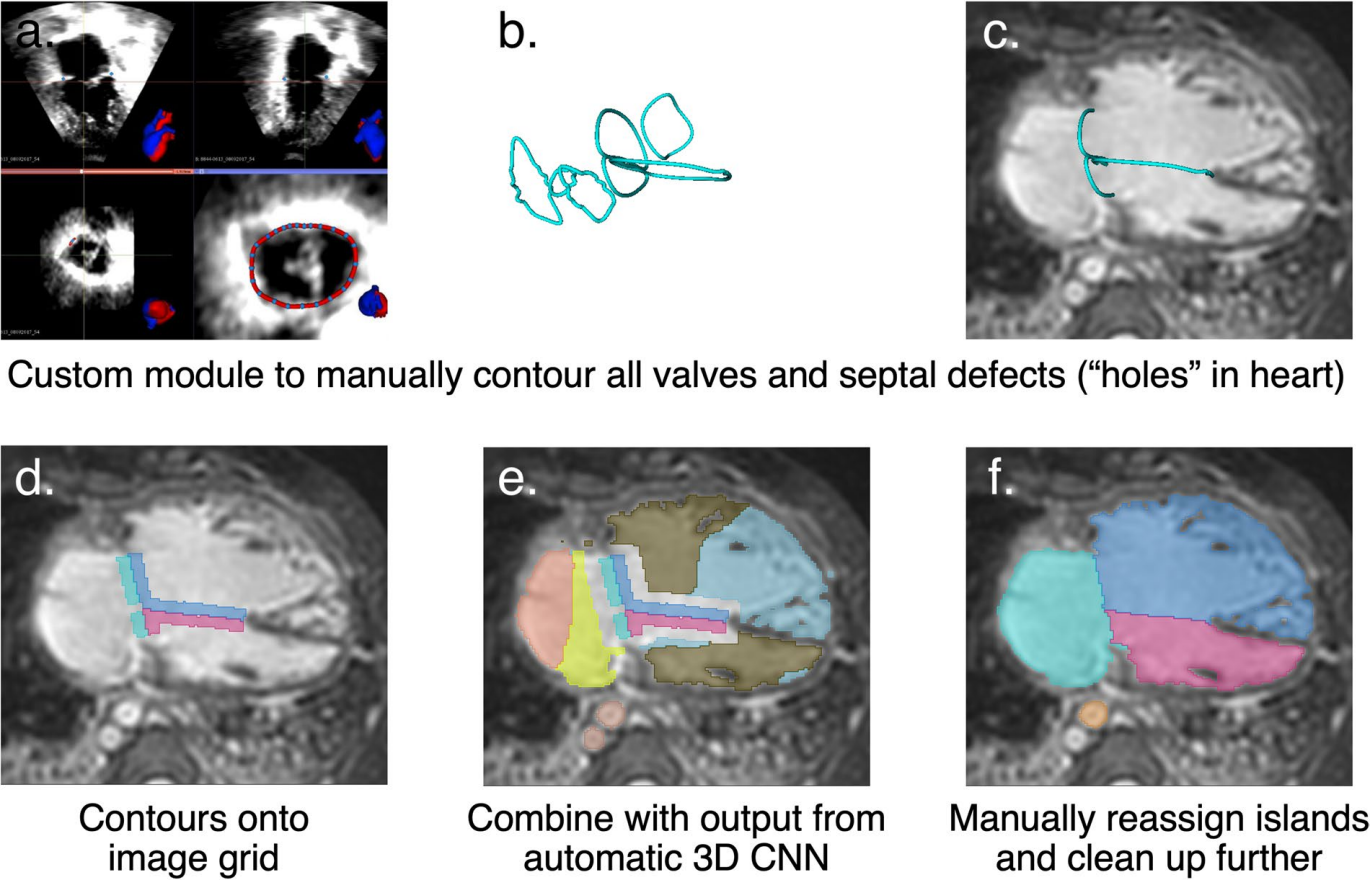
Ground truth whole-heart segmentations of the 40 new cases were created using a pipeline that merged manual annotations with outputs from a neural network ensemble trained on the 20 whole-heart segmentations from the HVSMR dataset.

The 40 new images were segmented using a custom semi-automatic pipeline that leveraged the new whole-heart segmentations of the 20 HVSMR scans. An overview is shown in Fig. 2.

First, trained raters (again, graduate and undergraduate students in medical image analysis, computer science and the life sciences) used a 3D Slicer module (SlicerHeart, https://github.com/SlicerHeart/SlicerHeart), originally designed for valve contouring in echocardiography^17^, to annotate roughly planar contours separating the 8 heart structures. Specifically, we used the “Valve Annulus Analysis” and “Valve Segmentation” modules. Raters had access to the list of heart defects for each case, as extracted from the cardiology codes, for help in forecasting which structures would be present, their approximate locations, and their connectivity to other structures.

Separately, an ensemble of four 3D U-Net^30^ convolutional neural networks was trained on the 20 cropped, intensity-normalized and manually-segmented HVSMR images. The 20 images were split into 4 training datasets as in 4-fold cross-validation. The model architecture had 4 levels, with 24 learned channels at the first level, a doubled number of features at each subsequent level, 3 × 3 × 3 maxpooling after the first level, and 2 × 2 × 2 maxpooling after the second and third levels^31^. Each model had approximately 3,600,000 learnable parameters. Data augmentation included random affine transformations, left-right and anterior-posterior flips (helpful due to dextrocardia and other cardiac malpositions in CHD), nonlinear transformations, constant intensity shifts, and additive Gaussian noise. All implementations were done using Keras (http://keras.io) with a Tensorflow backend (http://tensorflow.org). The loss function was a categorical cross-entropy loss with spatially-varying weights that address class imbalance and more strongly penalize errors near ground truth segmentation borders^31^. Model parameters were optimized using adadelta for 2000 epochs with a learning rate of 0.001 and a batch size of 1. Each of the four models produces a probability map over 9 classes (8 foreground structures plus background). For each new image, the segmentation inferred by the ensemble was created by averaging the probability maps from the four models and computing an *argmax*. Segmentations were post-processed to retain the largest connected component for each structure (or two largest connected components for cases with bilateral SVC or hepatics labeled as IVC).

This network did not perform very well, since it was trained on such a small dataset. For example, a subsequent study^31^ found that, using a similar model architecture and training scheme, the accuracy after training on segmentations of the 20 HVSMR whole-heart segmentations and testing on the 40 new subjects yielded an average dice score of 87.7 ± 14.6 for “mild”/“moderate” test subjects but only 64.6 ± 31.9 for “severe” test subjects.

Nevertheless, once combined with the manually contoured interfaces described above, segmentation cleanup via island relabeling and further painting or erasing was easier than manual segmentation from scratch. For details, see Table 3. A first pass was performed by trained raters (graduate and undergraduate students in medical image analysis, computer science and the life sciences), and the protocol was completed by the supervising annotator (D.F.P.). Note that the most difficult task, namely annotating the interfaces between structures, remained completely manual. In addition, the boundaries between each structure and the surrounding myocardium or vessel/chamber wall were carefully inspected and manually adjusted as necessary, avoiding bias towards the U-Net output. The time required is highly dependent on the complexity of the case. We estimate that our workflow requires approximately 4–8 hours per 3D CMR image, versus approximately 8–16 hours for purely manual segmentation.

**Table 3:** Protocol followed to manually correct automatic segmentations from an ensemble of four 3D U-Nets^30^, using manually contours separating the 8 heart structures.

**1. Superimpose contours onto image grid**
The contours were superimposed onto the image grid using SlicerHeart’s “Valve Segmentation“ module^17^. Two label maps were created for each contour: one above the contour (Scale = 90, Top distance = 3, Top scale = 100, Bottom distance = 0, Bottom scale = 0) and one below the contour (Scale = 90, Top distance = 0, Top scale = 0, Bottom distance = 3, Bottom scale = 100). The Scale parameter was decreased if the resulting contour segmentation did not remain within the the blood pool. All of the label maps (two per contour) were then manually merged and renumbered, creating a single label map representing manual contours on the image grid as shown in Fig. 2d.
**2. Merge contour segmentations and neural network segmentations**
A custom 3D Slicer python script merged the contour segmentation with the neural network output. In a normal heart, the resulting label map has 14 unique labels: 6 contour labels (surrounding the mitral valve, tricuspid valve, aortic valve, pulmonary valve, SVC insertion and IVC insertion) and 8 structure labels from the neural network (LV, RV, LA, RA, AO, PA, SVC and IVC). Some hearts have additional contour labels corresponding to VSDs or ASDs. The script automatically produces gaps surrounding each contour label, which will be filled in step 3 v) below. The result is depicted in Fig. 2e.
**3. Manual editing**
The aim of this step is to adjust the labels originating from the neural network, producing segmentations as in Fig. 2f. i) Click to remove extraneous floating islands (“Segment Editor“ module → “Islands“ effect → “Remove selected island“). ii) The neural network’s labeling of blood pool vs. myocardium is usually quite good, but it typically does not accurately split the blood pool into its chamber and vessel components. Click to relabel any connected components of the neural network that are mislabeled in their entirety (“Segment Editor“ module → “Islands“ effect → “Add selected island“). iii) Handle dark inhomogeneity artifacts: In the case of dark artifacts surrounding implanted stents, the supervising annotator (D.F.P.) manually segmented the vessel by interpolating between the visible ends on either side of the artifact and observing faint contours where possible. iv) Adjust the boundaries of each structure to create a final manually-edited segmentation (“Segment Editor“ module → “Paint“ effect, using “Editable intensity range“ for borders at the myocardium or a vessel wall). v) Remove gaps: A custom 3D Slicer python script filled the gaps surrounding each original contour label (the script uses “Segment Editor“ module → “Watershed“ effect).
**4. Define “optional zones“ for vessel segmentations**
i) Ensure that all vessel segmentations are as long as is optionally allowed, as described in Table 2 (“Segment Editor“ module → “Paint“ effect, using “Editable intensity range“). ii) LA: manually cut each PV to require its stump only (“Segment Editor“ module → “Scissors“ effect → “Erase inside“ or “Segment Editor“ module → “Paint“ effect). iii) AO, PA, SVC and IVC: These must be cut twice, to define the (1) minimum required vessel extent, and (2) ground truth endpoint. Custom 3D Slicer python scripts cut each vessel using manually placed fiducials, manually placed slice planes, or automatically placed slice planes, as described in Table 2.

All steps were performed using 3D Slicer^29^.

After segmenting the 40 new scans, the whole-heart segmentations of the original 20 HVSMR images were re-reviewed to verify plane placement and segmentation boundaries, which were manually adjusted as needed. This aimed to mitigate potential annotation changes between the old and new scans.

#### Additional considerations for vessels

Results from a previous whole-heart segmentation challenge noted that fair evaluation can be problematic when the ground truth does not have standardized vessel lengths^18^. To avoid this, the ground truth segmentations of the AO, PA, SVC and IVC were created with consistent endpoints based on cardiac landmarks (see Table 2). Custom 3D Slicer python scripts were written so that the vessels could be appropriately cut using the relevant manually placed fiducials, manually placed slice planes, or automatically calculated slice planes. This process creates the best segmentations to use for model training.

**Fig. 3:**
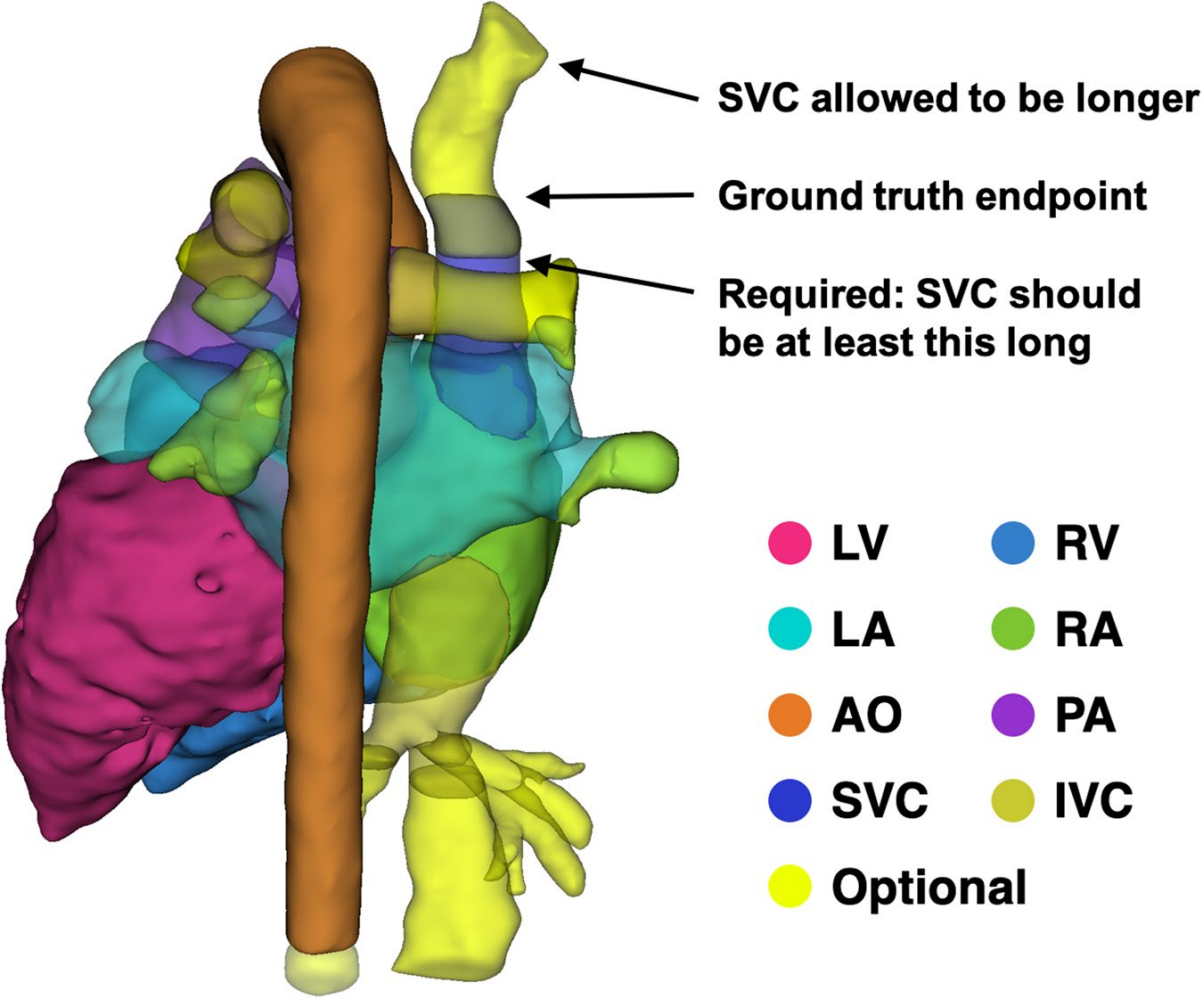
Example optional zones in the ground truth vessel segmentations for the AO, PA, SVC, IVC and LA pulmonary veins, with highlighted explanations for the SVC.

However, algorithms that produce vessel segmentations that are slightly too short or too long are just as useful clinically^18,32,33^. To address this, we established “optional zones” for the AO, PA, SVC, IVC and pulmonary veins (PVs, within the LA) that are derived from concrete landmarks and embody both minimum and maximum vessel lengths. More details are provided in Table 2, and an example is shown in Fig. 3. Again, 3D Slicer scripts were written to assist in implementing the segmentation protocol. Before model evaluation, the optional zones should be subtracted from both the ground truth segmentation and the model’s estimated segmentation, so that only the required regions of each are compared.

#### Final post-processing and review

Automated post-processing removed any small islands in the background or within individual segmentations, performed a mild smoothing, and ensured only one or two connected components per structure (when known). Finally, all 60 images were reviewed by a pediatric cardiologist (J.R.) to evaluate the accuracy of segmentation, and edited where necessary.

### Diagnosis information provided with each scan

Table 1 lists the applicable heart defects, prior surgeries, and coincident variants that were recorded for each scan. Under the advice of a pediatric cardiologist (J.R.), the presence or absence of each was determined based on the cardiology codes and written radiology report. Since the codes can be incomplete or refer to heart defects that had already been surgically corrected, the supervising annotator (D.F.P.), under the supervision of a pediatric cardiologist (J.R.), manually reviewed each image to verify that its list of defects, prior surgeries and coincident variants was correct and complete.

### Relationship to previous work

The first 20 images of HVSMR 2.0 come from the original HVSMR dataset, which was held as a challenge at MICCAI 2016 (http://segchd.csail.mit.edu)^21^. However, the segmentations we now provide for these images are completely different, with ground-truth annotations for 8 foreground structures instead of only 2.

The full dataset described here was used in previous image segmentation methods research from our group^31^. Our aim here is to make the data from this publication public, since there are currently no open datasets for whole-heart segmentation in CMR images from CHD patients. We also provide more detailed descriptions of the steps used to create, annotate and validate the dataset. We performed an additional step of validation and annotation revisions with a pediatric cardiologist (J.R.) for all 60 images. Finally, we provide additional clinical and technical information corresponding to each scan.

## Data Records

The dataset is available at figshare at 10.6084/m9.figshare.c.7074755.v2^27^, with this section being the primary source of information on the availability and content of the data being described. All images and segmentations are in the Neuroimaging Informatics Technology Initiative (NIfTI) format. Segmentations and endpoints files (which delineate the optional zones) include labels 1-LV, 2-RV, 3-LA, 4-RA, 5-AO, 6-PA, 7-SVC, and 8-IVC. The data is stored in three.zip files:

- *orig*: CMR images manually cropped at the chin without image normalization (*pat#_orig.nii.gz*), with corresponding whole-heart segmentations (*pat#_orig_seg.nii.gz*) and endpoints files (*pat#_orig_seg_endpoints.nii.gz*).
- *cropped*: CMR images manually cropped around the heart without image normalization (*pat#_cropped.nii.gz*), with corresponding whole-heart segmentations (*pat#_cropped_seg.nii.gz*) and endpoints files (*pat#_cropped_seg_endpoints.nii.gz*).
- *cropped_norm*: CMR images manually cropped around the heart after image normalization (*pat#_cropped_norm.nii.gz*), with corresponding whole-heart segmentations (*pat#_cropped_seg.nii.gz*) and endpoints files (*pat#_cropped_seg_end-points.nii.gz*).

We also provide two *.csv* files, *hvsmr2_clinical.csv* and *hvsmr2_technical.csv*, containing additional information for each scan. In each file, the *Pat* column gives the patient number, referring to the filenames listed above.

The *hvsmr2_clinical.csv* file contains demographic and clinical data. The *Age* column gives the age at scan time in years. Subjects under one year of age therefore have an age of ‘0’. The *Category* column indicates the degree of morphological malformations (“mild”, “moderate” or “severe”), as described in the “Subject categorization“ section above. The remaining columns include the detailed diagnoses, prior surgeries and coincident variants for each scan corresponding to Table 1. In each cell, X indicates that a condition applies.

The *hvsmr2_technical.csv* file contains image acquisition parameters and the dataset splits used in previous studies. The *TE*, *TR*, *FA* and *BW* columns show the echo time (ms), repetition time (ms), flip angle (°), and bandwidth (Hz) for the scan. The *HVSMR2016* column indicates whether the image was in the original HVSMR dataset, and if so, whether they were in the training split or the testing split. The *PaceMEDIA2022* column indicates whether the image was assigned to a cross-validation split (numbered 1–4) or the testing split in our group’s previous medical image analysis methods research that used this dataset^31^.

## Technical Validation

Subject selection, subject categorization, and manual segmentation were performed under the advice of pediatric cardiologists (T.G., Su.G., A.J.P), and underwent a final review for accuracy by a fourth pediatric cardiologist (J.R.). The segmentation process was supervised by an associate professor in pediatric radiology and cardiology (M.H.M.). Raters were supervised by an expert in medical image analysis with over 10 years’ experience at that time (D.F.P.). Training for the graduate and undergraduate students who performed annotations included education on CMR and CHD, written instructions, and frequent one-on-one consultation with D.F.P.

A quality control step was performed after each step of the segmentation process (D.F.P.). This included reviewing images for image quality, verifying the cardiology codes extracted from radiology reports, manually checking and editing the contours that were manually placed to separate cardiac structures, and manually checking and editing the whole-heart segmentations. All segmentations were first reviewed by an associate professor in pediatric radiology and cardiology (M.H.M.) and then again by a pediatric cardiologist (A.J.P for the 20 HVSMR images and J.R. for all).

## Usage Notes

Users should decide whether they want to use images that have been cropped around the heart and/or intensity normalized, and use the data in the corresponding folder as described in the “Data Records” section. We do not recommend mixing data between the *orig*, *cropped* and *cropped_norm* directories. Note that all images have a unique size and resolution, and typically must be resampled before model training.

When training models, users should use the provided whole-heart segmentations as the ground truth. However, during evaluation, the optional zones for vessels within the endpoints files should be considered. The optional zone segmentations should be subtracted from *both* the ground truth and the predicted segmentation (in one-hot representations) before computing a segmentation score, so that only the required regions are compared.

Users are free to use the metric of their choosing to evaluate segmentation accuracy. We recommend the Dice score (a widely accepted metric of volume overlap) and the Hausdorff distance (either the maximum Hausdorff distance or the 95th percentile, to provide a performance measure in millimeters, which is useful for clinicians). Note that some structures may be missing in the ground truth (e.g., in the case of single ventricle or common atrium).

Users can split the 60 cases into training, validation and testing datasets at their discretion. All segmentations are openly provided because we want to enable research in interactive segmentation, which may be necessary for challenging cases and for which there is no established biomedical challenge protocol. We do provide the splits used in our previous research^31^. This allows direct comparison between different works that use the same splits, while also providing flexibility to users to make the most sensible choices for their own research.

Our previous work^31^ found that segmentation neural networks typically perform well on both “mild” and “moderate” cases but are significantly challenged by the “severe” cases. We encourage authors to similarly report their results on “mild”/“moderate” and “severe” cases separately. In particular, networks may encounter challenges in distinguishing between left and right in severe cases with heterotaxy, dextrocardia, single ventricle, or common atrium. If a network mistakenly assigns the LA label to the RA, or the LV to the RV (or vice versa), dice scores will be significantly decreased.

Additional background on segmentation for congenital heart disease can be found in the Ph.D. thesis of Danielle F. Pace^5^, from which this paper was adapted.

## Data Availability

No custom code has been made available.
